# Effect of oral intake of royal jelly on endothelium function in hemodialysis patients: study protocol for multicenter, double-blind, randomized control trial

**DOI:** 10.1186/s13063-021-05926-x

**Published:** 2021-12-20

**Authors:** Kojiro Ohba, Yasuyoshi Miyata, Takeaki Shinzato, Satoshi Funakoshi, Kanenori Maeda, Tomohiro Matsuo, Kensuke Mitsunari, Yasushi Mochizuki, Tomoya Nishino, Hideki Sakai

**Affiliations:** 1 Department of Urology, Nagasaki University Hospital, 1-7-1 Sakamoto, Nagasaki, 852-8501 Japan; 2 Shinzato Clinic Urakami, 3-20 Mori-machi, Nagasaki, 852-8104 Japan; 3 Nagasaki Jin Hospital, 5-1 Kozen-machi, Nagasaki, 850-0032 Japan; 4 Maeda Clinic, 587-2 Shinden-machi, Shimabara, 855-0043 Japan; 5 Second Department of Internal Medicine, Nagasaki University Hospital, 1-7-1 Sakamoto, Nagasaki, 852-8501 Japan

**Keywords:** Royal jelly, Atherosclerosis, Hemodialysis, Endothelial function, ESCs, Oxidative stress, Angiogenic activity

## Abstract

**Background:**

Hemodialysis (HD) is a common renal replacement therapy for patients with renal failure. Cardiovascular and cerebrovascular diseases are known to shorten survival periods and worsen the quality of life of HD patients. Atherosclerosis is a major cause of vascular diseases, and various factors such as abnormality of lipid metabolism and increased macrophage activity, oxidative stress, and endothelial dysfunction are associated with its pathogenesis and progression. Further, endothelial stem cells (ESCs) have been reported to play important roles in endothelial functions. Royal jelly (RJ) affects atherosclerosis- and endothelial function-related factors. The main aim of this trial is to investigate whether oral intake of RJ can maintain endothelial function in HD patients. In addition, the effects of RJ intake on atherosclerosis, ESC count, inflammation, and oxidative stress will be analyzed.

**Methods:**

This will be a multicenter, prospective, double-blind, randomized controlled trial. We will enroll 270 participants at Nagasaki Jin Hospital, Shinzato Clinic Urakami, and Maeda Clinic, Japan. The participants will be randomized into RJ and placebo groups. The trial will be conducted according to the principles of the Declaration of Helsinki, and all participants will be required to provide written informed consent. The RJ group will be treated with 3600 mg/day of RJ for 24 months, and the placebo group will be treated with starch for 24 months. The primary endpoint will be the change in flow-mediated dilation (FMD), a parameter of endothelium function, from the time before treatment initiation to 24 months after treatment initiation. The secondary and other endpoints will be changes in FMD; ESC count; serum levels of vascular endothelial cell growth factor, macrophage colony-stimulating factor, 8-hydroxydeoxyguanosine, and malondialdehyde; the incidence of cardiovascular diseases, cerebrovascular diseases, and stenosis of blood access; and safety.

**Discussion:**

This trial will clarify whether oral intake of RJ can maintain endothelial function and suppress the progression of atherosclerosis in HD patients. In addition, it will clarify the effects of RJ on ESCs, oxidative stress, and angiogenic activity in blood samples.

**Trial registration:**

The Japan Registry of Clinical Trials jRCTs071200031.  Registered on 7 December 2020.

## Background

Hemodialysis (HD) is used as renal replacement therapy for the control of uremic syndrome in patients with renal failure. With the development of dialysis medicine, including devices, dialysates, and drugs, the survival period and quality of life (QoL) of HD patients have improved. However, various HD-related complications, especially cardiovascular and cerebrovascular diseases, shorten the survival periods and worsen the QoL in HD patients [1, 2]. Many factors, such as hypertension, hyperlipidemia, and uremia, are associated with the pathogenesis of vascular damage by complex mechanisms [3]. Furthermore, there is general agreement that atherosclerosis plays a crucial role in the development of vascular diseases and results in increased mortality among HD patients [4, 5]. Thus, an understanding of the detailed pathological mechanism of atherosclerosis and the development of therapeutic approaches for such pathological conditions are essential for improving the survival and QoL of these patients.

The pathophysiological mechanism of atherosclerosis has been well-studied worldwide, and many investigators suggest that the following four factors are strong determinants of the occurrence and development of atherosclerosis: (1) abnormal lipid metabolism, including oxidation of low-density lipoprotein (LDL) cholesterol, is an important step in the occurrence of atherosclerosis [6]; (2) differentiation and activation of macrophages play crucial roles in lipid homeostasis in atherosclerotic lesions [7]; (3) dysfunction of endothelial cells and injury to the arterial intima lead to cholesterol accumulation and formation of foam cells from infiltrated macrophages [7, 8]; and (4) inflammation and oxidative stress strongly regulate such pathological processes by a coordinated system [8, 9]. In addition to these representative atherosclerosis-related factors, several other factors are associated with the pathogenesis and development of atherosclerosis. For example, endothelial stem cells (ESCs) play an important role in vascular repair, especially when endothelial damage is severe [10]. Moreover, macrophage colony-stimulating factor (MCSF), which is a strong stimulator of macrophage differentiation and activity, is associated with atherosclerosis under various pathological conditions, including HD [11, 12]. Interestingly, MCSF has been reported to be associated with the differentiation, migration, and secretory capacity of ESCs [13–15]. Oxidative stress is also induced by the differentiation, survival, and activity of ESCs [16, 17]. However, the detailed clinical role of ESCs in the repair of vascular function in HD patients has not been studied.

Royal jelly (RJ) is a natural bee product that is widely used as a health food and supplement worldwide because of its anti-inflammatory, antioxidative, antibacterial, and anticancer effects [18, 19]. In addition, RJ has been reported to be associated with vasorelaxation and anti-hypertensive effects via vascular endothelial cell activity, angiogenesis, and nitric oxide production at the molecular level [20–23]. Moreover, several in vitro studies have shown that RJ affects the secretion of inflammation-related cytokines from activated macrophages [24, 25]. Unfortunately, little information is available on the association between RJ administration and inflammation-related cytokines in humans. However, oral intake of RJ can suppress anticancer agent-induced adverse events by regulating inflammation-related cytokines [26].

Therefore, in addition to its use in traditional medicine, RJ is administered as a modern therapeutic agent for various diseases, such as hypertension, hypercholesterolemia, and malignancies [19, 27]. However, a direct correlation between RJ and MCSF in humans has not been established. Interestingly, we found that the serum levels of MCSF changed after the oral intake of RJ in renal cell carcinoma patients treated with tyrosine kinase inhibitors (unpublished data). Based on these facts, RJ was speculated to affect various atherosclerosis-related factors, including inflammation, oxidative stress, endothelial function, and ESC activity. Therefore, this trial will be conducted to verify our hypothesis that oral intake of RJ is effective in improving vascular endothelial function, and consequently, suppressing the risk of cardiovascular and cerebrovascular diseases via regulation of inflammation, oxidative stress, and the activities of macrophages and ESCs in chronic HD patients.

## Methods

### Study design

**Fig. 1:**
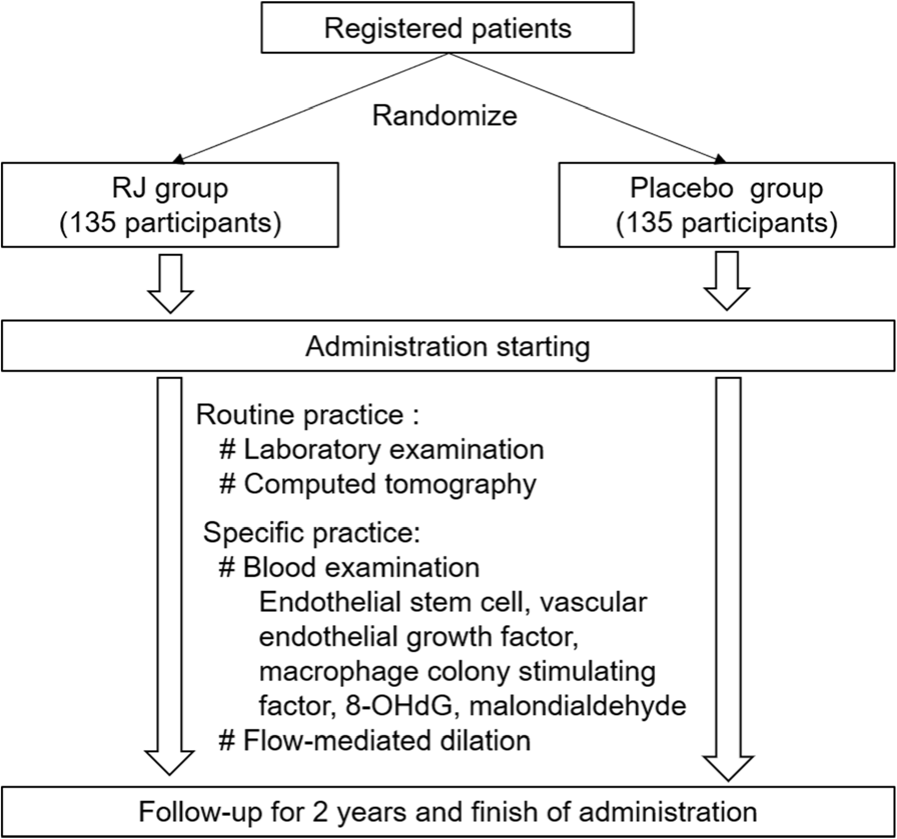
Flow chart of our study

**Fig. 2:**
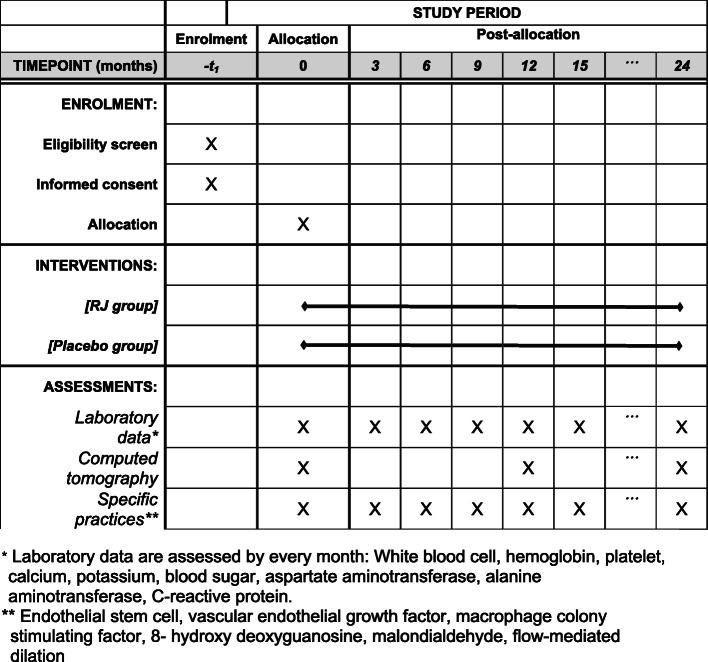
Timeline for all study patients

This clinical trial will be conducted in a multicenter, randomized, double-blind, placebo-controlled setting. The study is planned to investigate the superiority of RJ compared to placebo. Three institutions in Japan (Shinzato Clinic Urakami, Maeda Clinic, and Nagasaki Jin Hospital) will participate in this trial. Our RJ is the same as the commercial supplement manufactured in Yamada Bee Farm, and the recommended dosage of this supplement is 3600 mg/day. Therefore, in this trial, HD patients will be divided into two groups: the RJ group, which will be treated with 3600 mg/day of RJ for 24 months, and the placebo group, which will be treated with starch for 24 months. Both RJ and placebo were provided by the Institute for Bee Products and Health Science (Okayama, Japan). The flow chart and timeline of this study are shown in Figs. 1 and 2. The trial has been approved by the certified institutional review board of Nagasaki University Hospital (no. CRB7180001), and the protocol has been registered in the University Hospital Medical Information Network (UMIN000020152) and The Japan Registry of Clinical Trials (jRCTs071200031). In case of protocol modification, the contents of the change will be shown to relevant parties after approval by our IRB. There was no additional collection and use of participant data and biological specimens in ancillary studies. There will be no post-trial care. The individual data will not be disclosed to sponsor, participants, healthcare professionals, the public, and other relevant groups. On the other hand, analyzed data of all participants will be disclosed to all of these groups via publication and public-relation magazine. The full protocol, participant-level dataset, and statistical code will not be disclosed.

Consent about the planned storage of the biological samples will be obtained by the same consent form in this trial. However, we stated in the form that additional consent or opt-out would be necessary for the biological samples to be used for further analyses.

### Randomization

Randomization will be performed by an independent employee of Nagasaki University Hospital using the envelope method, and medical investigators at each institute will be responsible for allocating patients. In short, eligible patients will be randomly assigned in a 1:1 ratio using a computerized random number generator, and the allocation information will be placed in sealed envelopes. Each patient will be assigned a unique number to protect their personal information. The randomized list will be managed by only an independent data manager and will be concealed from all investigators and patients. All information regarding patient selection will remain confidential and unknown to all investigators, including outcome assessors and statisticians, until the completion of the trial. Emergent unblinding will not be possible in this trial.

### Inclusion criteria

The inclusion criteria are as follows.

1. Patients undergoing continuous hemodialysis because of chronic renal failure.
2. Patients who are expected to survive for more than 24 months after the treatment.
3. Patients with a performance status of 0–2 [28].
4. Patients aged 20 years and higher.
5. Patients who provide written informed consent prior to participating in the trial.
6. Patients with an understanding that all examinations, procedures, and treatments associated with HD will be performed per the provisions of medical care in Japan and that there is no compensation for symptoms, disease, and disorders in this trial.

Regarding inclusion criteria number 3, the scale developed by the Eastern Cooperative Oncology Group will be used to evaluate the performance status [28]. This scale is commonly used in patients with cancer; however, we used it because of the two following reasons: (1) there is no simple and useful scale to evaluate the performance status in patients with hemodialysis, and (2) it was previously used in patients with chronic dialysis [29].

### Exclusion criteria

The exclusion criteria are as follows.

1. Patients who already consume RJ or original bee food products.
2. Patients who plan to consume RJ during the trial period.
3. Patients with severe vascular diseases that need treatment.
4. Patients allergic to RJ or original bee food products.
5. Patients with a history of asthma or atopic dermatitis.
6. Pregnant or lactating patients.
7. Patients who are judged as unsuitable for inclusion in the trial by the principal investigator or co-investigators.

### Primary endpoint

The primary endpoint is the change in FMD before treatment and 24 months after treatment initiation.

### Secondary and other endpoints

The secondary and other endpoints are as follows.

1. FMD at 0, 3, 6, 9, 12, 15, 18, and 21 months after treatment initiation.
2. Aortic calcification index (ACI) at 0, 12, and 24 months after treatment initiation.
3. ESC count in the blood at 0, 3, 6, 9, 12, 15, 18, 21, and 24 months after treatment initiation.
4. Serum levels of vascular endothelial growth factor (VEGF), MCSF, 8-hydroxydeoxyguanosine (OHdG), and malondialdehyde (MDA) at 0, 3, 6, 9, 12, 15, 18, 21, and 24 months after treatment initiation.
5. Incidence of cardiovascular diseases, cerebrovascular diseases, and stenosis of blood access during the 24 months.
6. Adverse events of oral intake of RJ.

### Follow-up

The patients will visit the hospital 3–4 times per week for maintenance HD. The frequency of HD in a week will be determined by medical judgment based on laboratory data, clinical practice, and patient wishes. Similarly, decisions about the contents of HD will be made according to each institution’s policy independent of this trial. There are no relevant concomitant care and interventions that are permitted or prohibited during the trial.

### Sample size

Unfortunately, there are no reports on changes in FMD due to oral intake of RJ in HD patients. In addition, temporal changes in FMD during HD sessions are unclear. There is general agreement that RJ is a safe food, and our study design poses an extremely low risk to patients as the only other invasive intervention will be the collection of a blood sample (20 ml) every 3 months. Considering these aspects, the proposed sample size was calculated according to the expected agreement rates (60%) of patients, which is the object of this trial. Finally, 135 patients will be enrolled in each of the two groups. For recruitment, the posters that showed purpose, method, risk, and cost are displayed. All authors will obtain written informed consent from potential trial participants or authorized surrogates after oral explanation and providing the explanatory documents. The consent form is original, and it has been approved by our IRB.

We also calculated the proposed sample size according to a previous paper on FMD in HD patients and a meta-analysis of changes in FMD associated with the oral intake of another supplement (magnesium) [30, 31]. In short, 112 patients will be enrolled in each group (a total of 224 patients) with an alpha error rate of 0.05 and a power of 0.8. This is because incomplete data may be available for some patients (approximately 20% of the patients), and patient dropouts may occur before the completion of the trial. Finally, we plan to enroll 135 patients in each group. Regarding such processes, we have had repeated discussions with several biostatisticians. In addition, our decision on sample size was reviewed twice by IRB members, including other biostatisticians. Finally, this policy was approved by IRB because of limited information about the influence of RJ on FMD and the high safety of RJ.

### FMD measurement

FMD measurements will be performed by professionally trained investigators using the UNEX EF-18VG system (UNEX Corporation, Nagoya, Japan). FMD measurements for the brachial artery will be performed by an experienced cardiologist. Patients’ arms will be immobilized in the extended position, and the brachial artery will be scanned longitudinally 3–5 cm above the antecubital fossa. Baseline measurements for the brachial artery will be recorded, and the cuff will be inflated to 200 mmHg (or 50 mmHg higher than the systolic blood pressure) for 5 min to induce forearm ischemia. Subsequently, the cuff will be deflated, and the diameter of the brachial artery will be measured 60 s after deflation. FMD is defined as the percent change in the diameter of the brachial artery from baseline to post-reactive hyperemia. As a result, FMD will be obtained as a continuous variable. All measurements will be performed by a single investigator who will be blinded to the clinical details.

### ACI measurement

ACI will be measured according to our previous report [11]. In short, the percentage area of the aortic circumference occupied by calcified material will be measured in each sequential consecutive 10-mm slice in non-contrast computed tomography (CT), and the mean percentage of all slices will be calculated for each patient. ACI will be determined by two investigators in separate institutes, and the average of the two values will be used for analysis.

### ESC count

The ESC count will be determined using flow cytometry as previously reported [32]. Fresh peripheral blood mononuclear cells will be obtained by density gradient centrifugation, and cell counting will be performed immediately. The cells will be stained with the following antibodies: Brilliant Violet (BV) 421 mouse anti-human CD34 (IgG1, к), phycoerythrin (PE) CD133 (clone W6B3C1), and Alexa Fluor 637 mouse anti-human CD309 (VEGF-2) antibody. The following isotype control antibodies will be used: BV 421 mouse IgG1, к, PE mouse IgG1, к (clone MOPC-21), and Alexa Fluor (clone MOPC-21) antibodies. After surface staining for 30 min at 4 °C in the dark, the cells will be washed with phosphate-buffered saline solution. Surface-stained cells will be then subjected to fluorescence-activated cell sorting (FACS) using a BD LSRFortessa^TM^ flow cytometer. The ESC frequency will be defined as the number of cells per 10^6^ events for each sample. Based on the surface expression of CD34, CD133, and CD309, ESCs will be determined by flow cytometry and differentiated into four subpopulations, namely CD34^+^, CD34^+^CD133^+^, CD34^+-^CD309^+^, and CD34^+^CD133^+^CD309^+^ cells. These four cell subpopulations will be counted as ESCs as described previously [32].

### Enzyme-linked immunosorbent assay

Serum levels of VEGF (R&D Systems, Inc.; MN), MCSF (R&D Systems, Inc.; MN), 8-OHdG (Japan Institute for the Control of Aging, Nikken Seil Co., Ltd., Shizuoka, Japan), and MDA (ELISAGenie, London, UK) will be determined using enzyme-linked immunosorbent assay (ELISA; R&D Systems, Inc.; MN).

### Cardiovascular diseases, cerebrovascular diseases, and stenosis of blood access

Regarding cardiovascular diseases, new onset of ischemic heart disease and arrhythmia requiring medical treatment will be recorded. With regard to arrhythmia, frequent arterial or ventricular premature beats, supraventricular or ventricular tachycardia, and atrial fibrillation will be evaluated. In addition, the frequencies of hypertension, hypotension, and new-onset arrhythmia will be recorded. Hypertension and hypotension are defined as systemic blood pressures > 160 mmHg and < 80 mmHg, respectively. Cerebrovascular diseases include cerebral hemorrhage, cerebral infarction, and subarachnoid bleeding. Stenosis of blood access is defined as new-onset stenosis requiring balloon dilation and/or surgery.

### Data collection and management

Patients will be identified by a specific number, which will be assigned by independent staff. All clinical data will be collected from patient records. All data and information about this trial will be protected by passwords, and two persons responsible for information control will be able to access the data. In addition, three independent investigators will check the data for accuracy. Data monitoring will be performed according to the research plan approved by the clinical research review board of Nagasaki University. In short, the data management committee consisting of the approved data and information managers in Nagasaki University Hospital who were independent of competing interests and sponsors will oversee the handling of data, including storing and sharing data, every 3 months. To promote participant retention and complete follow-up, information on patients’ conditions and the data will be totalized in the executive office in Nagasaki University Hospital every 3 months. Any outcome data will be collected and listed for participants who discontinue or deviate, at the time of the event. All individual data and biological specimens will be preserved at the Nagasaki University Hospital until analyses. There is no plan to use the residual samples.

### Safety management

If serious adverse events (SAEs) that are speculated to be associated with oral RJ intake, RJ administration will be discontinued. In case of the existence of a causal relation between SAEs and RJ, this study will be terminated. Judgments about RJ-related SAEs will be made by medical investigators at each institute and will be reported to the ethics committee within 24 h and to the coordinate centers within 3–5 days (3 working days). If mild adverse events occur, RJ administration will be discontinued until recovery. In this study, changes in drug dose will not be permitted. On the other hand, RJ administration and all examinations will be terminated in case of participant withdrawal. In addition, the principal investigator (YM) must decide to terminate the trial even if one investigator suggests early termination because of SAEs.

### Statistical analyses

The results of all examinations will be expressed as mean ± SD. To clarify the primary endpoint, the changes in the rates of FMD after treatment for 24 months (FMD for 24 months/baseline) will be compared between the RJ and placebo groups using the Student’s *t* test. Non-adherence and missing data will not be used in this analysis. A similar analysis will be performed to compare the changes in FMD at 6, 12, and 18 months between the two groups. For this analysis, missing data will be used according to the period of RJ administration. Other variables and data will be analyzed similarly at 6, 12, 18, and 24 months. There is no plan for any additional analyses. Permitted investigators (KO, YM, TM, KM) will have access to these interim results.

## Discussion

The QoL and survival of HD patients have been improving with developments in dialysis medicine, including dialysis machines, dialyzers, and agents. However, cardiovascular and cerebrovascular diseases remain the major and severe complications in HD patients [1, 2]. Therefore, we analyzed temporal changes in vascular function among HD patients because many investigators support the opinion that endothelial dysfunction plays a crucial role in the pathogenesis and development of atherosclerosis, and such pathological conditions are closely associated with vascular diseases [33–35].

In this clinical trial, the endothelial function will be evaluated by a non-invasive method, FMD evaluation, in HD patients treated with RJ and placebo. FMD is widely used to measure endothelial function under various pathological conditions [36–38]. In addition, to clarify the repair of vascular endothelium, we will also measure serum VEGF levels because VEGF is a major regulator of endothelial cell growth and migration [39]. The degree of vascular calcification will be assessed using ACI. Currently, various noninvasive methods, such as aortic pulse wave velocity and ultrasonographic assessment of intima-media thickness, are widely used to evaluate vascular conditions [40, 41]. In this study, we will determine the ACI to evaluate the status of blood vessels because abdominal CT is routinely performed every 6–12 months in HD patients at our hospital. In short, there will be no additional disadvantages in terms of participation in this trial.

ACI is considered a useful marker for evaluating and predicting vascular disease development, including calcification and atherosclerosis, under various pathological conditions [42–44]. Oxidative stress is one of the major risk factors for atherosclerosis in patients undergoing HD [45, 46]. Therefore, we will measure serum levels of 8-OHdG and MDA because these markers are commonly used to evaluate the status of oxidative stress in HD patients [47, 48].

We will also investigate changes in ESC count in blood samples that are attributable to oral RJ intake. Previous reports showed that ESCs play an important role in the repair of injured endothelium and the prognosis of vascular diseases, and this mechanism is regulated by a variety of different factors, such as angiogenesis, macrophage activity, and oxidative stress [10, 13–15, 17, 49, 50]. Therefore, to clarify the molecular mechanisms of changes in ESCs, the changes in serum levels of VEGF, MCSF, MDA, and 8-OHdG will be measured in a similar study population.

To our knowledge, this will be the first randomized trial to evaluate the influence of RJ intake on endothelial function in HD patients. In addition, no clinical trial evaluating changes in the ESC count in blood and serum levels of MCSF, oxidative stress-related markers, and angiogenesis-related factors due to oral intake of RJ has been conducted in a similar study population. We expect to confirm whether RJ can improve endothelial function in HD patients and clarify the detailed molecular mechanisms underlying such anti-atherosclerotic function.

## Trial status

The protocol version is 1.0, 18 September 2020. Participant recruiting started in December 2020. The completion of recruitment is anticipated to be on 7 December 2022. The completion of the final follow-up is anticipated to be on 31 December 2024.

## Data Availability

The data that support the findings of this study are available from the corresponding author upon reasonable request.

## Abbreviations

ACI: Aortic calcification index
BV: Brilliant Violet
CT: Computed tomography
ESCs: Endothelial stem cells
FACS: Fluorescence-activated cell sorting
FMD: Flow-mediated dilation
HD: Hemodialysis
LDL: Low-density lipoprotein
MCSF: Macrophage colony-stimulating factor
MDA: Malondialdehyde
OHdG: Hydroxydeoxyguanosine
QoL: Quality of life
PE: Phycoerythrin
RJ: Royal jelly
SAEs: Serious adverse events
VEGF: Vascular endothelial growth factor
VEGFR: Vascular endothelial growth factor receptor

## References

[1] Malik J (2018). Heart disease in chronic kidney disease -review of the mechanisms and the role of dialysis access. J Vasc Access.

[2] Toyama T, Kasama S, Sato M, Sano H, Ueda T, Sasaki T, Nakahara T, Higuchi T, Tsushima Y, Kurabayashi M (2019). Most important prognostic values to predict major adverse cardiovascular, cerebrovascular, and renal events in patients with chronic kidney disease including hemodialysis for 2 years. Cardiology.

[3] Longenecker JC, Coresh J, Powe NR, Levey AS, Fink NE, Martin A, Klag MJ (2002). Traditional cardiovascular disease risk factors in dialysis patients compared with the general population: The CHOICE study. J Am Soc Nephrol.

[4] Kato A, Takita T, Maruyama Y, Kumagai H, Hishida A (2003). Impact of carotid atherosclerosis on long-term mortality in chronic hemodialysis patients. Kidney Int.

[5] Fabbian F, Casetta I, De Giorgi A, Pala M, Tiseo R, Portaluppi F (2012). Stroke and renal dysfunction: Are we always conscious of this relationship?. Clin Appl Thromb Hemost.

[6] Prati F, Marco V, Paoletti G, Albertucci M (2020). Coronary inflammation: Why searching, how to identify and treat it. Eur Heart J Suppl.

[7] Chistiakov DA, Bobryshev YV, Orekhov AN (2016). Macrophage-mediated cholesterol handling in atherosclerosis. J Cell Mol Med.

[8] Gimbrone MA, García-Cardeña G (2016). Endothelial cell dysfunction and the pathobiology of atherosclerosis. Circ Res.

[9] Marchio P, Guerra-Ojeda S, Vila JM, Aldasoro M, Victor VM, Mauricio MD (2019). Targeting early atherosclerosis: A focus on oxidative stress and inflammation. Oxid Med Cell Longev.

[10] Bai X, Wang X, Xu Q (2010). Endothelial damage and stem cell repair in atherosclerosis. Vasc Pharmacol.

[11] Kihara T, Miyata Y, Furukawa M, Noguchi M, Nishikido M, Koga S, Kanetake H (2005). Predictive value of serum macrophage colony-stimulating factor for development of aortic calcification in haemodialysis patients: A 6 year longitudinal study. Nephrol Dial Transplant.

[12] Singhal A, Subramanian M (2019). Colony Stimulating Factors (CSFs): Complex roles in atherosclerosis. Cytokine.

[13] Nakano K, Adachi Y, Minamino K, Iwasaki M, Shigematsu A, Kiriyama N, Suzuki Y, Koike Y, Mukaide H, Taniuchi S, Kobayashi Y, Kaneko K, Ikehara S (2006). Mechanisms underlying acceleration of blood flow recovery in ischemic limbs by macrophage colony-stimulating factor. Stem Cells.

[14] Zhang Y, Adachi Y, Iwasaki M, Minamino K, Suzuki Y, Nakano K, Koike Y, Mukaide H, Shigematsu A, Kiriyama N, Li C, Ikehara S (2006). G-CSF and/or M-CSF Accelerate Differentiation of bone marrow cells Into Endothelial Progenitor Cells in vitro. Oncol Rep.

[15] Schäfer R, DeBaun MR, Fleck E, Centeno CJ, Kraft D, Leibacher J (2019). Quantitation of progenitor cell populations and growth factors after bone marrow aspirate concentration. J Transl Med.

[16] Case J, Ingram DA, Haneline LS (2008). Oxidative stress impairs endothelial progenitor cell function. Antioxid Redox Signal.

[17] Lam YT (2015). Critical roles of reactive oxygen species in age-related impairment in ischemia-induced neovascularization by regulating stem and progenitor cell function. Oxid Med Cell Longev.

[18] Pasupuleti VR, Sammugam L, Ramesh N, Gan SH (2017). Honey, propolis, and royal jelly: A comprehensive review of their biological actions and health benefits. Oxid Med Cell Longev.

[19] Khazaei M, Ansarian A, Ghanbari E (2018). New findings on biological actions and clinical applications of royal jelly: A review. J Diet Suppl.

[20] Izuta H, Chikaraishi Y, Shimazawa M, Mishima S, Hara H (2009). 10-Hydroxy-2-decenoic Acid, a major fatty acid from royal jelly, inhibits VEGF-induced angiogenesis in human umbilical vein endothelial cells. Evid Based Complement Alternat Med.

[21] Kawano Y, Makino K, Jinnin M, Sawamura S, Shimada S, Fukushima S, Ihn H (2019). Royal jelly regulates the proliferation of human dermal microvascular endothelial cells through the down-regulation of a photoaging-related microRNA. Drug Discov Ther.

[22] Tokunaga KH, Yoshida C, Suzuki KM, Maruyama H, Futamura Y, Araki Y, Mishima S (2004). Antihypertensive effect of peptides from royal jelly in spontaneously hypertensive rats. Biol Pharm Bull.

[23] Liang Y, Kagota S, Maruyama K, Oonishi Y, Miyauchi-Wakuda S, Ito Y, Yamada S, Shinozuka K (2018). Royal jelly increases peripheral circulation by inducing vasorelaxation through nitric oxide production under healthy conditions. Biomed Pharmacother.

[24] Kohno K, Okamoto I, Sano O, Arai N, Iwaki K, Ikeda M (2004). Royal jelly inhibits the production of proinflammatory cytokines by activated macrophages. Biosci Biotechnol Biochem.

[25] Sugiyama T, Takahashi K, Kuzumaki A, Tokoro S, Neri P, Mori H (2013). Inhibitory Mechanism of 10-hydroxy-trans-2-decenoic Acid (Royal Jelly Acid) Against lipopolysaccharide- and interferon-β-Induced nitric oxide Production. Inflammation.

[26] Miyata Y, Araki K, Ohba K, Mastuo T, Nakamura Y, Yuno T, Mukai Y, Otsubo A, Mitsunari K, Mochizuki Y, Sakai H (2020). Oral intake of royal jelly improves anti-cancer effects and suppresses adverse events of molecular targeted therapy by regulating TNF-α and TGF-β in renal cell carcinoma: A preliminary study based on a randomized double-blind clinical trial. Mol Clin Oncol.

[27] Miyata Y, Sakai H (2018). Anti-cancer and protective effects of royal jelly for therapy-induced toxicities in malignancies. Int J Mol Sci.

[28] Oken M, Creech R, Tormey D, Horton J, Davis TE, McFadden ET (1982). Toxicity and response criteria of the Eastern Cooperative Oncology Group. Am J Clin Oncol.

[29] Sičaja M, Pehar M, Đerek L, Starčević B, Vuletić V, Romić Ž (2013). Red blood cell distribution width as a prognostic marker of mortality in patients on chronic dialysis: a single center, prospective longitudinal study. Croat Med J.

[30] Ozkok A, Atas R, Cinar SA, Yilmaz A, Aktas E, Deniz G, Yildiz A (2017). CD133+ cells are associated with ADIPOCYTOKINES and endothelial dysfunction in hemodialysis patients. BMC Nephrol.

[31] Darooghegi Mofrad M, Djafarian K, Mozaffari H, Shab-Bidar S (2018). Effect of magnesium supplementation on endothelial function: A systematic review and meta-analysis of randomized controlled trials. Atherosclerosis.

[32] Fadini GP, Sartore S, Agostini C, Avogaro A (2007). Significance of endothelial progenitor cells in subjects with diabetes. Diabetes Care.

[33] Miglinas M, Cesniene U, Janusaite MM, Vinikovas A (2020). Cerebrovascular disease and cognition in chronic kidney disease patients. Front Cardiovasc Med.

[34] Peng R, Luo M, Tian R, Lu N (2020). Dietary nitrate attenuated endothelial dysfunction and atherosclerosis in apolipoprotein E knockout mice fed a high-fat diet: A critical role for NADPH oxidase. Arch Biochem Biophys.

[35] Zhao TC, Wang Z, Zhao TY (2020). The important role of histone deacetylases in modulating vascular physiology and arteriosclerosis. Atherosclerosis.

[36] Tomisawa T, Nanashima N, Kitajima M, Mikami K, Takamagi S, Maeda H, Horie K, Lai FC, Osanai T (2019). Effects of blackcurrant anthocyanin on endothelial function and peripheral temperature in young smokers. Molecules.

[37] de Camargo FCF, DeMoura JR, Cepeda FX, de Almeida CM, Nascimento RC, Fortes-Queiroz L (2020). Photobiomodulation by low-level laser therapy in patients with obstructive sleep apnea: Study protocol clinical trial (SPIRIT compliant). Med (Baltim).

[38] Zhang G, Fan Y, Qiu Y, Zhou Z, Zhang J, Wang Z, Liu Y, Liu X, Tao J (2020). Allisartan Isoproxil improves endothelial function and vascular damage in patients with essential hypertension: A Single-Center, open-label, randomized controlled trial. Adv Ther.

[39] Gomes de Almeida Schirmer B, Crucet M, Stivala S, Vucicevic G, da Silva Barcelos L, Vanhoutte PM (2020). The NO-donor MPC-1011 Stimulates angiogenesis and Arteriogenesis and Improves hindlimb ischemia via a cGMP-dependent Pathway Involving VEGF and SDF-1α. Atherosclerosis.

[40] Bartoli-Leonard F, Wilkinson FL, Schiro A, Inglott FS, Alexander MY, Weston R. Loss of SIRT1 in diabetes accelerates DNA damage induced vascular calcification. Cardiovasc Res. 2020;cvaa134. 10.1093/cvr/cvaa134. 10.1093/cvr/cvaa134 PMC7898956 32402066

[41] Machin DR, Auduong Y, Gogulamudi VR, Liu Y, Islam MT, Lesniewski LA (2020). Lifelong SIRT-1 overexpression attenuates large artery stiffening with advancing age. Age Ageing (Albany, NY).

[42] Kim DW, Hwang SY, Nam YJ, Kim D, Shin SJ, Yoon HE (2020). The combined prognostic significance of alkaline phosphatase and vascular calcification in patients with end-stage kidney disease. Nutr Metab Cardiovasc Dis.

[43] Nam YJ, Hwang SY, Kim DW, Kim D, Shin SJ, Yoon HE (2020). Sex-specific relationship between vascular calcification and incident fracture in patients with end-stage renal disease. Kidney Res Clin Pract.

[44] Schousboe JT, Vo TN, Langsetmo L, Adabag S, Szulc P, Lewis JR, Kats AM, Taylor BC, Ensrud KE (2020). Abdominal aortic calcification (AAC) and ankle-brachial index (ABI) predict health care costs and utilization in older men, independent of prevalent clinical cardiovascular disease and each other. Atherosclerosis.

[45] Rapa SF, Di Iorio BR, Campiglia P, Heidland A, Marzocco S (2019). Inflammation and oxidative stress in chronic kidney disease-potential therapeutic role of minerals, vitaminS and plant-derived metabolites. Int J Mol Sci.

[46] Ali NE, Kaddam LA, Alkarib SY, Kaballo BG, Khalid SA, Higawee A, AbdElhabib A, AlaaAldeen A, Phillips AO, Saeed AM (2020). Gum arabic (*Acacia Senegal*) augmented total antioxidant capacity and reduced C-reactive protein among haemodialysis patients in Phase II trial. Int J Nephrol.

[47] Satoh M, Yamasaki Y, Nagake Y, Kasahara J, Hashimoto M, Nakanishi N, Makino H (2001). Oxidative stress is reduced by the long-term use of vitamin E-coated dialysis filters. Kidney Int.

[48] Rangel-López A, Paniagua-Medina ME, Urbán-Reyes M, Cortes-Arredondo M, Alvarez-Aguilar C, López-Meza J (2013). Genetic damage in patients with chronic kidney disease, peritoneal dialysis and haemodialysis: A comparative study. Mutagenesis.

[49] Alexandru N, Safciuc F, Constantin A, Nemecz M, Tanko G, Filippi A, Dragan E, Bãdilã E, Georgescu A (2019). Platelets of healthy origins promote functional improvement of atherosclerotic endothelial progenitor cells. Front Pharmacol.

[50] Pelliccia F, Pasceri V, Moretti A, Tanzilli G, Speciale G, Gaudio C (2020). Endothelial progenitor cells predict long-term outcome in patients with coronary artery disease: Ten-year follow-up of the PROCREATION extended study. Int J Cardiol.

